# Genetic profiling in radiotherapy: a comprehensive review

**DOI:** 10.3389/fonc.2024.1337815

**Published:** 2024-07-26

**Authors:** Dino Rubini, Federico Gagliardi, Vittorio Salvatore Menditti, Luca D’Ambrosio, Paolo Gallo, Ida D’Onofrio, Antonio Rosario Pisani, Angela Sardaro, Giuseppe Rubini, Salvatore Cappabianca, Valerio Nardone, Alfonso Reginelli

**Affiliations:** ^1^ Department of Precision Medicine, University of Campania “L. Vanvitelli”, Naples, Italy; ^2^ Interdisciplinary Department of Medicine, University of Bari ‘Aldo Moro’, Bari, Italy

**Keywords:** radiotherapy, genomic profiling, radiogenomics, radiation oncology (RO), profiling

## Abstract

This comprehensive review explores the pivotal role of radiotherapy in cancer treatment, emphasizing the diverse applications of genetic profiling. The review highlights genetic markers for predicting radiation toxicity, enabling personalized treatment planning. It delves into the impact of genetic profiling on radiotherapy strategies across various cancer types, discussing research findings related to treatment response, prognosis, and therapeutic resistance. The integration of genetic profiling is shown to transform cancer treatment paradigms, offering insights into personalized radiotherapy regimens and guiding decisions in cases where standard protocols may fall short. Ultimately, the review underscores the potential of genetic profiling to enhance patient outcomes and advance precision medicine in oncology.

## Introduction

Radiotherapy (RT) plays a vital role in cancer treatment, aiming to deliver precise and effective radiation doses to tumor tissues while minimizing damage to surrounding healthy cells. However, the response to radiotherapy can vary significantly among cancer patients due to intrinsic biological differences. As advancements in genomics and molecular biology continue to unravel the intricacies of cancer biology, genetic profiling has emerged as a promising tool to tailor radiotherapy regimens to individual patients’ specific needs.

This comprehensive review delves into the diverse landscape of genetic profiling in radiotherapy, exploring its potential applications in various oncological settings. From predicting radiation toxicity to optimizing treatment outcomes in different cancer types, genetic profiling holds the key to unlocking the full potential of precision medicine in radiotherapy.

The review presents a detailed analysis of several key oncological settings where genetic profiling has shown significant promise. First, we investigate the role of genetic markers in predicting radiation toxicity, allowing clinicians to identify patients who may be at higher risk of adverse side effects from radiotherapy. Understanding the genetic factors influencing treatment-related toxicity is crucial for personalized treatment planning and ensuring better quality of life for cancer survivors.

Furthermore, this review extensively examines genetic profiling’s impact on radiotherapy strategies in breast cancer, lung cancer, esophageal cancer, rectal cancer, brain cancer, prostate cancer, and gynecological cancer. In each of these contexts, we delve into the latest research findings, identifying genetic signatures associated with treatment response, prognosis, and therapeutic resistance. This wealth of knowledge provides a foundation for designing tailored radiotherapy approaches that are uniquely suited to the genetic makeup of individual tumors.

Moreover, the review highlights the potential for genetic profiling to guide treatment decisions in cases where standard radiotherapy protocols may not suffice. By identifying genetic alterations associated with RT treatment resistance, clinicians can explore novel therapeutic avenues and combination strategies to enhance the effectiveness of radiotherapy.

In conclusion, the integration of genetic profiling in radiotherapy marks a transformative shift in cancer treatment paradigms. By dissecting the molecular underpinnings of individual tumors, genetic profiling empowers oncologists with unprecedented insights, enabling the delivery of targeted and personalized radiotherapy regimens. Through an in-depth analysis of various oncological settings, this review aims to shed light on the immense potential of genetic profiling in shaping the future of radiotherapy, with the ultimate goal of improving patient outcomes and advancing the field of precision medicine in oncology.

## Search strategy

In April 2023, a literature search was conducted to identify eligible studies on the combination of radiotherapy and genetic profiling. PubMed/MEDLINE and Embase electronic databases were utilized, employing the keywords “Radiotherapy AND genomic OR genetic profiling.” The search included original articles up to March 1, 2023.

The exclusion criteria were established to filter out non-original articles. This includes abstracts, editorials, book chapters, letters, congress communications, or posters. Non-English articles, papers unrelated to genomic profiling with radiotherapy, and studies that focus on genomic profiling with only chemotherapy were also excluded.

A total of 2952 studies were identified, and after title and abstract analysis, 313 papers meeting the specified exclusion criteria were selected. The publications, spanning from 2010 to 2023, exclusively comprised retrospective studies in the analysis. After full-text analysis, following the selection process, 50 papers were deemed eligible for the results analysis.

## Breast cancer

The management of breast cancer has undergone a notable transformation towards personalized treatment approaches, driven by advancements in genomic profiling. An array of genomic tests has surfaced, undergoing scrutiny to enhance risk assessment and inform treatment choices, particularly in systemic therapies. Many of these tests are currently undergoing investigation in the realm of radiotherapy as well, reflecting a comprehensive endeavor to optimize treatment strategies across various modalities.

Several genomic tests are currently available for breast cancer patients, such as the Prosigna^®^ assay, analyzing 50 genes, and the Oncotype Dx test, analyzing 21 genes and the MammaPrint that analyzes 70 genes.

Radiation therapy tailored to specific genetic signatures has emerged as a promising approach in breast cancer treatment. Several studies have examined the prognostic value of gene expression profiles in predicting locoregional recurrence (LRR), encompassing patients with diverse characteristics such as age, hormone receptor (ER)/HER2 status, and treatment modalities received. Notably, the exploratory substudy of the EORTC10041/BIG 03-04 MINDACT trial, involving 5360 patients treated with breast-conserving surgery and adjuvant radiotherapy, found that the 70-gene signature did not significantly correlate with LRR in a multivariable model (1). Similarly, Tramm et al. investigated the prognostic and predictive value of a seven-gene profile (DBCG-RT profile) in high-risk breast cancer patients undergoing mastectomy with or without post-mastectomy radiotherapy (PMRT), revealing no significant benefit from PMRT in patients identified with a low-risk profile (2). In contrast, the Adjuvant Radiotherapy Intensification Classifier (ARTIC), comprising 27 genes and patient age, demonstrated predictive validity for LRR in patients randomized to receive RT or no RT after BCS in the SweBCG91-RT trial (3). Patients with a low ARTIC-profile derived substantial benefit from RT. Additionally, the same group tested the predictive utility of a 16-gene signature called Profile for the Omission of Local Adjuvant Radiation (POLAR) was validated in various cohorts, indicating a decreased risk of LRR with RT in high-risk patients (4). Moreover, the genomic-adjusted radiation dose (GARD) has emerged as a radiosensitivity biomarker that integrates gene-expression-based radiation-sensitivity index and linear quadratic model, aiding in predicting therapeutic response to RT in breast cancer and other malignancies (5). However, GARD is applicable only to conventionally fractionated RT and does not account for genomic and microenvironmental heterogeneity. With this approach, Nolan et al. (2022) used the Genomic Adjusted Radiation Dose (GARD) model and RNA-seq to assess radiosensitivity in breast and prostate cancer patients and showed how overall dose and fractionation, in correlation with mRNA transcription, can influence the choice of radiotherapy treatment towards a personalized treatment (6).

Also, molecular signatures for ductal carcinoma *in situ* (DCIS) have emerged as potential tools to guide RT indications, offering prognostic insights and predicting RT benefit, thus influencing adjuvant treatment decisions toward RT de-escalation, with different approaches (7–11).

Other Investigators have focused their research on the prediction of response analyzing the molecular profile (12, 13), single mutations such as PIK3CA (14), combination therapies (15) or proton therapy (16), whereas other Investigators focused on machine learning approaches (17) and hypoxia gene signatures (18).

Regarding side effects, in 2012, Raabe et al. conducted a retrospective study to identify associations between specific single nucleotide polymorphisms (SNPs) and the risk of severe erythema after breast-conserving radiotherapy (19). In addition to assessing the presence of any genetic characteristics, Adam J. Webb et al. through the REQUITE study evaluated the correlation of presence of late breast atrophy and time of RT treatment by examining the circadian rhythm of patients. The study shows that women with the PER3 gene, the T/T genotype of rs2087947, show the least atrophy when treated at 3:30 pm, while the A/A genotype of rs11545787 in RASD1 shows the same effect in the morning. Opposite homozygotes of both genes have the highest rates of atrophy when treated at 15:30. In contrast, the G/G genotype of rs1801260 in CLOCK shows the lowest rates of atrophy with afternoon treatment, despite previous discordant studies. Heterozygotes for these three genes show no significant change in atrophy rates over time, with a similar risk of atrophy at 10:30 for all genotypes (20).

Personalized radiotherapy based on genetic signatures offers a promising pathway to improve the treatment of breast cancer, allowing greater accuracy in predicting locoregional recurrence and the radiosensitivity of patients. This approach aims to optimize therapy by tailoring radiation doses to individual genetic characteristics, while reducing side effects. However, the efficacy of different genetic signatures varies, and the applicability of these methodologies requires further studies to be completely validated. Continued research is essential to overcome the challenges of genomic variability and tumor heterogeneity to make these tools clinically relevant on a large scale.

All the studies are summarized in **Table 1**.

**Table 1:** Summary of studies investigating the use of genetic profiling in breast cancer radiotherapy.

First Author	Year	Aim	Results
Alaeikhanehshir	2024	Risk of LRR in patients with early-stage breast cancer treated with BCS.	LRR at 8 years of 3.2% after BCS, the 70-gene test was not predictive of LRR.
Tramm	2014	Identify genes predicting benefit of RT, receive or not receive PMRT.	Identification of the genes HLA-DQA, RGS1, DNALI1, hCG2023290, IGKC, OR8G2 and ADH1B catalogued in the DBCG-RT genetic profile in patients with very low risk of LRR and no benefit from PMRT.
Sjöström M	2019	Develop a genomic tool to select the optimal RT strategy.	By analysing the transcriptome (the set of all RNA transcripts) of the patients’ primary tumours, a genetic classifier called ARTIC was developed. ARTIC was found to be prognostic for LRR and predictive for the benefit of RT.
Sjöström M	2023	Develop and validate a genomic profile to predict the use of radiotherapy in women with low-risk breast cancer.	The POLAR genomic signature based on 16 genes (AGR2, B4GALT1, CLDN7, EZR, GNG11, JUN, MMP11, PKIB, PRPS1, PSMD10, SH3BP5, SLC16A3, SLC7A11, SPP1, TNNT1, UBE2E1) can identify patients with a low risk of LRR despite not receiving RT.
Scott	2016	Identifying the optimal RT dose can be determined using a molecular signature of patient-specific radiation sensitivity.	Patients with high GARD had better distant metastasis-free survival than those with low GARD.
Nolan	2022	Use of the GARD model to assess radiosensitivity in breast cancer	Increased probability of tumour control by increasing the overall effective biological dose without increasing normal toxicity
Alvarado M	2015	Clinical utility of the 12-gene DCIS score™ in guiding the recommendation for RT	Recommendations for radiotherapy decreased from 73% to 59.1%, suggesting a more personalized approach to treatment.
Ouattara	2023	Risk stratification using molecular signatures to assess the impact of adjuvant RT after BCS in DCIS.	The Oncotype^©^ DX DCIS test and DCISionRT can discriminate between high and low risk women, suggesting a trend towards de-escalation of RT.
Wärnberg	2021	Validation of DCISionRT^®^ to predict the benefit of RT for women with DCIS	Patients were stratified into high (DS > 3) and low (DS ≤ 3) risk groups. The benefit of RT was greatest in women with a DS score greater than 2.8, suggesting that DCISionRT may help to tailor adjuvant treatment strategies for DCIS.
Vicini	2023	Identification of a novel biosignature for DCIS patients at high risk of LRR after BCS and adjuvant RT.	DCISionRT made it possible to distinguish between patients at low, high LRR risk, suggesting therapeutic approaches.
Shah	2021	Using DCISionRT^®^ to evaluate adjuvant RT in DCIS patients	Before the test, RT was recommended for 69% of patients; however, after the test, a change was made to the RT recommendation for 42% based on risk class.
Langlands FE	2013	Predict the response to radiotherapy in different breast cancer subtypes based on molecular profiles.	Expression levels of Holliday junction recognition protein (HJURP) mRNA and high cytoplasmic expression of peroxiredoxin-I correlated with increased LRs after RT
Mahmoud O	2016	Molecular classification of breast cancer and its association with RT response.	Octotype triple-negative breast cancer (TNBC) is associated with a poorer prognosis due to its radioresistance.
Bernichon E	2017	Identify predictive molecular biomarkers of LRR	PIK3CA mutation was associated with a lower risk of LRR, consistent with studies suggesting its role in impacting radiosensitivity.
Brassesco	2018	*In vitro* effects of two PLK1 inhibitors (BI 6727 and GSK461364) in combination with RT.	Significant reduction in clonogenicity and sensitization of cells to RT.
Bravatà	2018	Comparing molecular responses induced by irradiation with electrons and protons	Both types of ionizing radiation activate specific pathways in a radiation-dependent manner. Each cell line activates similar molecular networks in response.
Tabl AA	2019	A Machine Learning Approach identifying genes that may predict treatment class and serve as potential biomarkers.	The proposed model demonstrated high accuracy in patient classification and could tailor therapy based on gene expression.
Yan D	2022	Identification of a model that integrates immune and hypoxic gene signatures to identify radiosensitive and radioresistant patients.	Biomarkers that may predict response to RT include: PAK6, which is involved in radiosensitivity; PLXND1 and SEMA7A, genes involved in cancer cell growth; and SERPINE1, an oncogene linked to drug resistance; S100A4 and TGFBI may also be potential biomarkers. Genes linked to the spread and outlook of breast cancer.
Raabe	2012	Association of specific SNPs with risk of severe erythema after RT.	There was no significant association found between SNPs and the risk of erythema for all patients. However, in patients with small breasts, the TGFB1 gene was associated with erythema, whereas the XPD gene showed an association in patients with large breasts.
Webb	2022	Correlation of time of day’s RT and incidence of acute and late toxicity	Reduce atrophy by treating in mornings in PER3 genotype patient

LRR, locoregional recurrence; BCS, breast-conserving surgery; SNP, single nucleotide polymorphisms; PMRT, postmastectomy radiotherapy; RT, radiotherapy; ARTIC, Assessment of Radiotherapy Individualization in Breast Cancer; POLAR, Profile for the Omission of Local Adjuvant Radiation; GARD, Genomic-Adjusted Radiation Dose; DCIS, Ductal Carcinoma In Situ; DS, Decision Score; PLK1, Polo-like kinase I.

## Lung cancer

Lung cancer is the most common type of cancer and causes the most cancer-related deaths worldwide, with 1.6 million deaths annually. The most common type of lung cancer is non-small cell lung cancer (NSCLC). Treatment for NSCLC depends on the patient’s physical condition and the stage of the cancer. Surgery is the preferred treatment for early stages, but radiotherapy is used in 70% of lung cancer cases (21).

As technology advances, there are more strategies to improve lung cancer treatment. Radiogenomics in lung cancer is a rapidly evolving field that aims to understand the role of genes and genetic variations in the response to cancer treatments.

Its accurate study in relation to radiotherapy could lead to personalized treatments for patients with improved prognosis and reduced toxicity. The details of the analyzed studies are summarized in **Table 2**.

**Table 2:** Summary of studies investigating the use of genetic profiling in lung cancer radiotherapy.

First Author	Year	Aim	Results
Quian Huang	2015	The review discusses the role of genes involved in the mechanism of radiation-induced damage, especially RPand RIET. The study focuses on the potential value of SNP models to identify lung cancer individuals at risk for RP and RIET.	Among the several genes investigated, correlations have been found for RIET and SNPs of TGF-β1 especially for the CT/TT genotypes (≥ grade 3 toxicity). For RP, beside TGF-β1, mutations in ATM gene have showed significant correlation (especially ATM-111 G > A and ATM-126713 G>A polymorphisms)
Lara-Guerra Humberto	2016	Using a viral vector (adenovirus), the authors restored function of p53 in order to demonstrate a better response to both Chemotherapy and Radiotherapy	Using an intratumoral delivery of Ad5CMV-p53 in patients receiving sequential therapy of cisplatin and p53 gene therapy enhances p53 expression. This was confirmed via tumor biopsies showing an increase of 79% in cell apoptosis. Same showed in p53 injections with concomitant radiotherapy. In biopsies obtained 3 months after therapy, no viable tumor was observed in 63% of cases.
Anakura Mai	2019	Analyze differences in radiosensitivity between cells with EGFR mutations and those without mutations.	Using different cell lines, EGFR mutant cell lines were more sensitive to low dose- and low fraction sized-irradiation while no radiosensitivity differences between EGFR mutant and wild-type groups at high doses. This may suggest that hypofractionated irradiation is likely to achieve tumor control regardless of EGFR status.
Pu Zhou	2020	Reveal the change in genome mutation, RNA transcript of tumor cells, and TME after SBRT in lung tumor samples collected from 10 patients with NSCLC	all 10 tumor samples showed new mutations after radiation. Moreover, PD-L1 (CD274) expression in the TME significantly increased after SBRT than before radiation. However, no increase in CD8+ T-cell and NK-cell infiltration in the TME was found.
Shaverdian N	2022	Prove that TMB and mutations in genes associated with radiation sensitivity can predict for disease control in patients treated with CRT and durvalumab	high-TMB predicts for improved progression-free survival and local-regional control. Compared to patients with low-TMB, patients with high-TMB had improved PFS and less LRF: 24-month PFS: 66% vs 27%
Gao J	2022	Clarify the prognostic role of radiotherapy-related autophagy genes (RRAGs) in lung adenocarcinoma (LUAD)	three autophagy genes have been identified: SHC1, NAPSA, and AURKA. They seem to be strongly linked to immune cell infiltration. However no standard radiation schedule was used.

RP, radiation pneumonitis; RIET, radiation-induced esophageal toxicity; SNP, Single Nucleotide Polymorphism; TME, tumor microenvironment; SBRT, stereotactic body radiotherapy; NSCLC, non-small cell lung cancer; TMB, tumor mutational burden; CRT, chemoradiotherapy; PORT, post-operative radiotherapy.

In addition to the potential to personalize radiotherapy, genetic profiling may also help predict the prognosis of patients following RT treatment.

Shaverdian et al. examined the impact of tumour mutational burden (TMB) and genetic alterations associated with radiation response on post-operative radiotherapy (PORT) outcomes in resected NSCLC.

Patients with mutations in radiation resistance genes (KEAP1/NFE2L2/STK11/PIK3CA) had significantly more local-regional failures than those without so this suggests that this subset of patients may benefit minimally from PORT and may require different oncological strategies (22).

In NSCLC, genes related to autophagy caused by radiation (RRAGs) may have a prognostic role at the genomic level. Gao et al. compared control and irradiation-resistant cells and identified 3 genes (SHC1, NAPSA and AURKA) associated with prognosis and radioresistance in patients undergoing lung RT. These 3 genes may regulate the tumor microenvironment (TME) by modulating the immune environment and the effect of cell proliferation; indeed, radioresistance is partly due to high levels of autophagy (23).

To improve the effectiveness and prognostic value of radiation therapy in NSCLC patients, it may be useful to examine the expression of these genes that may be potential future therapeutic targets.

Zhou et al. revealed the changes in genome mutation and TME after stereotactic body radiotherapy (SBRT) in NSCLC tissues. The study found that RT increased PD-L1 expression in the TME and induced gene neo-mutation in tumor cells (24).

This result suggests that a second biopsy after RT may be necessary for PD-L1 negative tumours to evaluate mutations that could increase receptor expression after radiation therapy. It is crucial to identify potential molecular changes that could provide new systemic therapeutic options for the patient.

Another strategy to use genetic profiling as a tool in the fight against NSCLC is gene therapy, which involves the introduction of therapeutic genes or the correction of defective genes to target and mitigate identified genetic abnormalities.

Lara-Guerra et al. showed that transferring the p53 gene using retroviral or adenoviral vectors can induce apoptosis and tumor regression in certain cell lines and animal models of NSCLC and can increase the sensitivity of tumor cells to chemotherapy and radiotherapy.

Furthermore, the transfer of the TUSC2 gene via liposomal nanovesicles and the use of EGFR inhibitors may be a promising strategy to overcome resistance to targeted drugs.

If proven effective *in vivo*, this could lead to new therapeutic strategies (25).

Radiation pneumonitis (RP) and radiation-induced esophageal toxicity (RIET) are the most common adverse effects of lung RT. It is essential to have a clear understanding of these potential complications and to implement effective management strategies to prevent the patient from developing acute RP at the end of RT treatment or from failing to complete the entire RT cycle without interruption due to high esophageal toxicity (26, 27).

For this reason, Huang et al. examined the SNPs of several genes related to radiation-induced damage in lung cancer patients undergoing RT. The study identified several key genes, such as ATM, RAD51, TGF-β1, VEGF and others, that may influence radiation sensitivity and resistance, suggesting that SNPs in these genes may be used as biomarkers to predict the risk of RP and RIET in lung cancer patients and to personalize radiotherapy treatment (28).

The potential to use genetic profiling to personalize and modify the dose of radiotherapy has also been suggested by Anakura et al. Their *in vitro* study investigated whether EGFR mutation status influences the response of NSCLC cells to radiotherapy.

The research showed that cells with mutations in EGFR were more sensitive to radiotherapy than wild-type cells, especially at low doses and fractions.

The genetic profile of EGFR may predict tumour response to radiotherapy and this information can be used to optimize radiotherapy schedules by reducing the total dose, which can also lower the risk of potential toxicity (29).

The combination of genetic profiling and radiotherapy is emerging as a promising and dynamic approach in the fight against NSCLC. Comprehensive tumor analysis not only enables personalized treatment strategies, but also facilitates the identification of potential genetic markers that influence treatment response and prognosis. With the ability to tailor radiation doses based on genetic knowledge, there is greater potential to optimize therapeutic outcomes and minimize adverse effects.

Integrating genetic information into therapeutic decision-making is a critical step towards more effective, personalized and innovative strategies in the ongoing quest to improve outcomes for patients with NSCLC.

## Gastrointestinal cancer

### Esophagus

Currently, radiation therapy is considered a crucial treatment for cancer of the esophagus. Although radiotherapy has made significant progress, its side effects and resistance still present a major challenge in the clinical setting. It is therefore essential to identify new molecular biomarkers that can improve the radiosensitivity of esophageal cancer. This would further improve the prognosis and treatment outcomes for people diagnosed with esophageal cancer (30). Chen H et al. conducted a study to identify miRNA-mRNA regulatory networks affecting radiosensitivity in esophageal cancer, using The Cancer Genome Atlas (TCGA) and Gene Expression Omnibus (GEO) databases. They constructed a miRNA-mRNA regulatory network based on 47 miRNA-mRNA interactions, which included 21 miRNAs and 21 mRNAs. The study concluded that miR-132-3p/CAND1/ZDHHC23 and miR-576-5p/AHR are two key molecular pathways associated with esophageal cancer radiosensitivity (31). Another study suggesting the role of miRNAs is that of Lynam-Lennon N et al. published in the 2012 Journal of Molecular Medicine volume. This study demonstrates a role of miR-31 in the modulation of radioresistance, in fact, the expression of miR-31 was significantly reduced in patients showing a poor histomorphological response to neoadjuvant CRT, while the expression of DNA repair genes regulated by miR-31 was significantly increased. Irradiation was performed at a dose rate of 3.25 Gray (Gy)/min (32). In 2015, Hsu FM et al. conducted a study of 37 patients with esophageal squamous cell carcinoma (ESCC) to determine the effectiveness of circulating messenger RNA (mRNA) profiling in predicting complete response to treatment. The study followed patients for an average of 38 months and analyzed the three most differentially expressed circulating mRNAs (CCNL1, FAM84B and SEPT4) by quantitative RT-PCR. The research also identified FAM84B as a novel biomarker for predicting pathological response to neoadjuvant CRT. The study revealed that patients with a greater reduction in FAM84B mRNA expression in peripheral blood after CRT were more likely to achieve a complete response to treatment (33). Finally, another study conducted by Lynam-Lennon N et al. in 2017 analyzed the role of miRNAs in the treatment of EAC. The study used both *in vitro* models of radioresistant and chemoresistant EAC and biopsies from EAC patients to determine that miR-17-5p plays a significant role in the modulation of radioresistance *in vitro*. The study also found that miR-17-5p expression is significantly reduced in EAC tumors from patients who have a poor response to neoadjuvant CRT. Downregulation of miR-17-5p could be a mechanism supporting radioresistance in the EAC. The study used both *in vitro* models of radioresistant and chemoresistant EAC and biopsies from EAC patients to determine that miR-17-5p plays a significant role in the modulation of radioresistance *in vitro*. The study also found that miR-17-5p expression is significantly reduced in EAC tumors from patients who have a poor response to neoadjuvant CRT. Downregulation of miR-17-5p could be a mechanism supporting radioresistance in the EAC. The study used both *in vitro* models of radioresistant and chemoresistant EAC and biopsies from EAC patients to determine that miR-17-5p plays a significant role in the modulation of radioresistance *in vitro*. The study also found that miR-17-5p expression is significantly reduced in EAC tumors from patients who have a poor response to neoadjuvant CRT. Downregulation of miR-17-5p could be a mechanism supporting radioresistance in the EAC.

Radiotherapy is a mainstay in the treatment of esophageal cancer, but its effectiveness is limited by side effects and tumour resistance. Identifying new molecular biomarkers is crucial to improve the radiosensitivity of esophageal tumours, potentially improving prognosis and treatment outcomes. This approach could enable more targeted and personalized therapies, reducing treatment resistance and increasing the probability of complete therapeutic responses. Research on miRNA and mRNA is opening new avenues to better understand the mechanisms of radiosensitivity and develop more effective strategies against esophageal cancer.

All the studies are summarized in **Table 3**.

**Table 3:** Summary of studies investigating the use of genetic profiling in esophageal cancer radiotherapy.

First author	Year	Aim	Result
Chen H	2022	Identify miRNA - mRNA regulatory networks impacting radiosensitivity in esophageal cancer.	miR-132-3p/CAND1/ZDHHC23 and miR-576-5p/AHR molecular pathways play a crucial role in the radiosensitivity.
Lynam-Lennon N	2012	role of miR-31 in the modulation of radioresistance	In patients not responded to neoadjuvant CRT, there was a marked decrease in miR-31 expression, accompanied by an increase in the expression of miR-31-regulated DNA repair genes.
Hsu FM	2016	Examine changes in circulating mRNA profiles before and after neoadjuvant treatment.	3 distinctly expressed circulating mRNAs, namely CCNL1, FAM84B and SEPT4. FAM84B recognized as a novel prediction biomarker of pathological response to neoadjuvant CRT.
Lynam-Lennon N.	2017	Evaluate miR-17-5p as a potential biomarker of CRT sensitivity.	MiR-17-5p levels are markedly lower in tumors of patients with oesophageal adenocarcinoma showing poor response to neoadjuvant chemoradiotherapy.

CRT, Chemoradiotherapy.

### Rectum

Standard treatment for patients with UICC stage II/III rectal cancer is preoperative chemoradiotherapy (CRT) and total mesorectal excision (34). While this treatment approach is effective in controlling tumors and increasing outcomes rates, it also has some disadvantages such as mortality, morbidity, and long-term complications that can impact quality of life. It has become apparent that preoperative CRT does not have the same level of benefit for all patients with rectal cancer. While some patients may respond well, others may show no signs of cancer cells in the surgical specimen. Radiotolerance of subsistence of cancer stem cells could be the main obstacle in the treatment of this disease (35). In 2014, González-González M and colleagues conducted an analysis using fluorescence *in situ* hybridization (FISH) to examine 51 different DNA sequences located on chromosomes and chromosomal regions that are commonly altered in locally advanced rectal cancers. The study included a total of 76 tissue samples, 45 of which were obtained before treatment and 31 after. The researchers found a significant association between response to radiochemotherapy before surgery and rectal cancers showing 1p chromosome alterations. Posttreatment samples often showed additional gains in chromosomal regions 8q, 13q, and 20q, regardless of the degree of response to adjuvant radiochemotherapy (36). In 2011 a study was conducted by Cecchin E. et al. and published in The Pharmacogenomics Journal, which evaluated 238 rectal cancer patients undergoing fluoropyrimidine-based chemoradiotherapy (RT) in a neoadjuvant setting. The study analyzed 25 genetic polymorphisms in 16 genes related to treatment-associated pathways. Results showed that two polymorphisms, hOGG1-1245C > G and MTHFR-677C > T, were linked to a pharmacogenetic profile that predicts tumor regression grade (TRG) in a multivariate analysis. The hOGG1-1245C > G polymorphism can affect radiosensitivity, while the MTHFR-677C > T polymorphism is involved in the action of fluoropyrimidines. Patients with at least one allele of the variant were less likely to achieve a TRG ≤ 2 (37). In a study published in 2020 in the International Journal of Radiation Biology, Anuja K and colleagues found that HCT116-derived radioresistant cells have higher levels of the Shelterin RAP1 protein. The team also found that silencing RAP1 leads to increased DNA damage, resulting in increased sensitivity to radiation. These results suggest that increased RAP1 expression may help promote radioresistance in CRC cells by altering DNA damage and CSC phenotype (38). In 2018, Huang EY and colleagues conducted a study to examine the relationship between carcinoembryonic antigen (CEA) and rectal cancer. The study included 104 patients with stage II or III rectal cancer, undergoing post-operative radiotherapy (PORT) with radiation doses between 45 and 54.6 Gy. CEA levels were measured preoperatively. Previous research has shown that CEA can stimulate M2 macrophage differentiation, leading to radioresistance. The study found that pretreatment CEA levels ≥10 ng/mL were a significant risk factor for overall survival (OS), distant metastases (DM), and local recurrence (LR) after PORT for cancer of the rectum (39). In 2019, Kamran SC et al. conducted a study of locally advanced rectal adenocarcinoma tumors in 17 patients undergoing neoadjuvant CRT. The study involved integrated whole exome/transcriptome sequencing and analysis of tumor immune infiltrate before and after exposure to the CRT. Of the 17 patients, 9 were classified as responders (R) and 8 as non-responders (NR). The study found that there were no significant differences in TMB before and after exposure to CRT. Furthermore, there was no correlation between neoantigen burden before or after CRT and response to treatment. However, NR tumors had a higher incidence of coexisting mutations of KRAS and TP53, known as the KP genotype, than R tumors. Importantly, all tumors showed microsatellite stability throughout the study (40). In September 2022, Chang YK. et al. conducted a study to determine whether oncogenic and tumor suppressor mutations play a role in the different outcomes of patients with rectal cancer undergoing neoadjuvant chemoradiotherapy (nCRT). The study involved 29 patients who provided 29 rectal cancer samples. The results showed that BRAF, SMAD4 and TP53 gene mutations were more commonly found in patients with poor response to treatment than in those with complete response (41). In summary, based on the existing literature, none of the biomarkers can be considered reliable for an accurate prediction of the outcome of neoadjuvant CRT in rectal cancer. It is worth noting that epigenetic factors may also play a role, and further investigation is needed. SMAD4 and TP53 were more commonly found in patients with poor response to treatment than in those with complete response.

We can therefore conclude that despite the good efficacy of neoadjuvant radiochemotherapy treatments, significant disadvantages related to mortality, morbidity and long-term complications persist. Furthermore, the response to chemoradiation therapy varies among patients, with some responding well and others showing resistance to treatment. The presence of radiotolerant cancer stem cells is one of the main obstacles. Research has identified various genetic and molecular markers, such as genetic polymorphisms and specific proteins, that may influence the response to treatment. However, no biomarker has proven completely reliable in predicting the outcome of neoadjuvant chemoradiation therapy. Further studies are needed to better understand the mechanisms of resistance and to develop more personalized therapeutic approaches, including the consideration of epigenetic factors.

All the studies are summarized in **Table 4**.

**Table 4:** Summary of studies investigating the use of genetic profiling in rectal cancer radiotherapy.

First author	Year	Aim	Result
Garcia-Aguilar J	2011	Identify biomarkers associated with resistance to neoadjuvant chemoradiotherapy in rectal cancer	KRAS mutation, CCND1 G870A polymorphism, and MTHFR C677T polymorphism have been associated with non-pCR.
González-González M	2014	To examine the correlation between clonal evolution pathways within tumors and their resistance to neoadjuvant radiochemotherapy before surgery	A striking difference was observed between the efficacy of CRT and the presence of 1p chromosomal abnormalities in rectal carcinomas. In several cases, gains in chromosomal regions 8q, 13q, and 20q were found in posttreatment samples, regardless of response to adjuvant radiochemotherapy
Cecchin E	2010	A professional study was conducted to identify a pharmacogenetic profile that predicts the extent of tumor regression in patients with rectal cancer after receiving neoadjuvant treatment. The study aims to provide valuable insights into personalized medicine for patients with rectal cancer.	There are two specific gene variations that have been linked to a pharmacogenetic profile that predicts the extent of tumor regression (TRG). These variants are known as hOGG1-1245C>G, which may affect radiation sensitivity, and MTHFR-677C>T, which plays a role in fluoropyrimidine function. Patients who have one of these genetic variants are less likely to achieve a TRG ≤ 2. It is important to consider these genetic factors when evaluating the potential effectiveness of a treatment plan.
Anuja K	2019	role of RAP1 in the maintenance of the resistance phenotype and acquired stemness in radioresistant cells.	Increasing RAP1 expression levels can markedly increase radioresistance in CRC cells, regulating DNA damage and CSC phenotype. This finding highlights the crucial role of RAP1 in promoting radioresistance in CRC cells.
Huang EY	2018	identify carcinoembryonic antigen (CEA) as a marker of radioresistance in rectal cancer.	In the presence of M2 macrophages, CEA can lead to radioresistance. A CEA level of 10 ng/mL or greater prior to PORT treatment for rectal cancer has been found to pose a significant risk for overall survival, distant metastases, and local recurrence.
Kamran SC	2019	Identify characteristics associated with the response to treatment at the molecular level.	The cohort showed no notable changes in TMB before and after exposure to CRT. Furthermore, no correlation was found between neoantigen load before or after CRT and response to treatment. However, the researchers observed a higher frequency of co-mutations of KRAS and TP53 (KP genotype) in NR tumors than in R ones.
Chang YK	2022	To evaluate whether oncogenic and tumor suppressor gene mutations are involved in the differential outcomes of rectal cancer patients undergoing nCRT.	The group that did not respond well had a higher incidence of three genetic mutations, namely BRAF, SMAD4 and TP53, than the group that showed a complete response
Conde-Muíño R	2015	Examine the most commonly sought-after biomarkers to predict outcome of neoadjuvant CRT in patients with LARC	Neoadjuvant chemoradiotherapy provides some level of pathologic response in approximately 40-60% of patients with rectal cancer. However, there is currently no reliable approach to anticipate patients’ reaction to neoadjuvant treatment.

## Glioblastoma

Glioblastoma multiforme (GBM) is a highly aggressive malignant brain tumor. It is the most common primary brain tumor, accounting for 48.6% of all central nervous system malignancies. GBM is highly aggressive, and patients have a low median overall survival (OS) of only 15 months.

The 2016 fourth WHO classification of gliomas is based on histopathological criteria:

- IDH wildtype glioblastoma, which accounts for 90% of cases, typically develops in individuals in their 60s.
- IDH-mutant glioblastoma, which accounts for 10% of cases, usually develops in younger patients and has a better prognosis.
- Glioblastoma not otherwise specified (NOS);
- Glioblastoma not otherwise classified (NEC).

Standard therapy for GBM involves surgical resection, followed by chemoradiotherapy. This includes 6 weeks of temozolomide (TMZ) in combination with RT, followed by adjuvant TMZ for 5 days every 28 days for six cycles.

TMZ is a molecular alkylating agent that directly damages DNA with radiosensitizing properties. The efficacy of TMZ increases in the presence of a methylated MGMT promoter in GBM cells, which is the strongest positive prognostic marker (42, 43).

Radiation resistance is one of the major challenges in treatment of Brain cancer due to the high chance of tumor relapse and poor clinical outcomes. Genomic information offers great potential for optimization of cancer treatment, partly due to the rapidly decreasing sequencing costs we are seeing in the past few years.

Significant progress has been made in genetic profiling to understand the genomic basis of prognosis.

Fan F. et al. in 2021 found that the prognosis of glioblastoma (GBM) can be determined by seven differentially expressed genes (DEGs) 47, namely CLEC5A, HOXC6, HOXA5, CCL2, GPRASP1, BSCL2, and PTX3. These genes were verified in various GBM cell lines through real-time PCR. The risk scores derived from these DEGs were found to hold prognostic value regardless of other clinical factors, including IDH mutation status, and were inversely correlated with TP53 expression (44).

Additionally, pseudo-time analysis in neoplastic cells showed that the prognostic genes were linked with tumor proliferation and progression.

Gao et al. instead analyzing postoperative patients with GBM found that the gene expression profiles of protein-coding genes (PCG) and long non-coding RNA (lncRNA) could be associated with improved survival.

The PCG-lncRNA signature demonstrated a strong ability to predict survival and response to chemoradiation therapy with TMZ.

Risk scoring models were constructed, and the PCG-lncRNA signature was used to divide patients into high-risk or low-risk groups with significantly different survival rates.

This article suggests that new biomarkers could be used to predict prognosis and treatment outcomes (45).

Chanez et al. analyzed the recurrences of patients who had undergone surgery and concomitant CRT to identify possible genomic drivers of inevitable recurrence of glioblastoma IDHwt.

They found that low mRNA expression of Multiple PDZ Domain Crumbs Cell Polarity Complex Component (MPDZ) was significantly correlated with worse overall survival in both IDHwt and IDH mutated gliomas.

These results suggest that altering MPDZ may contribute to the systematic resistance of these tumors, opening new therapeutic possibilities (46).

Wang et al. researched whether genomic profiling could contribute to the radioresistance of GBM cells.

They analyzed gene expression in neoplasm samples before and after irradiation and identified 10 genes related to DNA metabolism that significantly up regulated in response to irradiation.

The POLQ, PRIM1, and RPA1 genes had the most significant impact on radioresistance. Suppression of these genes increased the radiosensitivity of tumor cells, while overexpression conferred increased resistance.

Therefore, these genes could be potential therapeutic targets to improve the efficacy of radiotherapy (47).

Genomic profiling can help identify new targets to improve GBM radiosensitivity.

It can identify specific molecular markers or genetic aberrations that affect tumor response to radiation.

Sun et al. investigated the role and mechanism of miR-153-3p in glioma radiosensitivity. They found that miR-153-3p is significantly reduced in radioresistant glioma samples and cell lines. However, its overexpression increases radiosensitivity by promoting apoptosis and reducing the activity of BCL2, an anti-apoptotic gene.

The authors conclude that miR-153-3p could be a potential therapeutic target to improve the effect of radiotherapy on GBM patients (48).

The integration of genomic profiling with radiation therapy is a significant step in the development of better therapeutic strategies for GBM.

Analyzing the tumor’s genetic landscape provides valuable insights into the molecular complexities that determine the aggressive nature of GBM. This information not only helps tailor therapeutic approaches but also has the potential to reveal new molecular targets that could improve the efficacy of radiotherapy.

All the studies are summarized in **Table 5**.

**Table 5:** Summary of studies investigating the use of genetic profiling in brain cancer radiotherapy.

First Author	Year	Aim	Results
Gao WZ	2018	PCGs and lncRNA signature with superior predictive power for GBM treatment outcome, especially in TMZ-chemoradiation treated patients.	PCGs and lncRNAs could be associated with improved survival. The PCG-lncRNA signature showed a strong ability to predict survival and response to chemoradiation therapy with TMZ.
Sun	2018	Investigate the role of miR-153-3p in radioresistance in glioma cells and its potential as a therapeutic target to enhance radiosensitivity.	Overexpression of miR-153-3p increases radiosensitivity by promoting apoptosis and reducing the activity of BCL2, an anti-apoptotic gene.
Wang C	2019	Investigate whether genomic profiling may contribute to the radioresistance of GBM cells.	Suppression of the POLQ, PRIM1 and RPA1 genes increased the radiosensitivity of tumour cells, while overexpression conferred increased resistance.Therefore, these genes could be potential therapeutic targets to improve the efficacy of radiotherapy.
Fan F	2021	Expression of genes CLEC5A, HOXC6, HOXA5, CCL2, GPRASP1, BSCL2, and PTX3 could serve as prognostic markers independently of other clinical factors.	expression of these genes induces tumour cell proliferation independently of other factors, including IDH mutation status.
Chanez	2022	Identifying possible genomic factors of unavoidable glioblastoma IDHwt recurrence in patients undergoing surgery and concomitant CRT	Low MPDZ mRNA expression was significantly correlated with worse overall survival in both IDHwt and IDH-mutated gliomas. These results suggest that MPDZ alteration may contribute to the systematic resistance of these tumours.

GBM, Glioblastoma; TMZ, Temozolamide; CRT, Chemoradiotherapy.

## Prostate cancer

According to researches, prostate cancer (PCa) continues to be the most prevalent form of cancer identified in men in the Western world (49, 50). Germline genetic testing and genomic testing have transformed the way prostate cancer patients are managed. Germline genetic testing identifies hereditary alterations, which are genetic changes that can be passed from one generation to the next. Detecting these alterations helps healthcare providers determine whether a patient is at an increased risk of developing prostate cancer due to inherited genetic mutations. This information is crucial for making informed decisions about screening, prevention, and treatment options. Genomic tests primarily focus on somatic alterations, which are genetic changes that occur within an individual’s tumor cells. These alterations may provide valuable information about the specific genetic mutations that drive the growth and progression of prostate cancer (51). Prostate cancer susceptibility is influenced by the BRCA1 and BRCA2 genes. Germline BRCA2 mutations have been associated with a higher risk of prostate cancer, increased mortality, and earlier age of diagnosis. BRCA1 mutations also increase the risk of prostate cancer, although to a lesser extent. Several genomic tests are available for prostate cancer patients, including the Oncotype DX Prostate test, which is a clinically validated 17-gene genomic test that provides a prostate genomic score (GPS) on a scale of 0-100 (52). This test measures the heterogeneous nature of prostate tumors and is performed on prostate tissue collected during the biopsy and the genomic information obtained facilitates optimal decision-making by the multidisciplinary team regarding the specific treatment.

It is a known fact that 40% of men aged 65 years or older undergo curative radiotherapy (RT). Of these, 10-30% are likely to experience biochemical recurrence. This highlights the significance of implementing effective therapeutic measures for patients with PCa (53). For patients undergoing definitive treatment or RT during the oligo/metastatic stages of disease, there is limited information available on the efficacy of genomic classifiers (GCs) in improving risk stratification after surgery. However, the use of GCs has shown promise in this regard. It is important to note that more research is needed to fully understand the benefits and limitations of GCs in these patient populations (54).

In 2018, JW and colleagues conducted a study to investigate the link between CD44 and radiation resistance. The results showed that inhibition of CD44 helped to increase the sensitivity of Cap cells to radiation. Researchers used a 160 kV photon linear accelerator, delivering a dose of 1.21 Gy/min for cellular irradiation. They created four cell lines, including two control groups and two irradiated groups. Tumor volumes in control and CD44 knockdown cell lines did not differ before irradiation. However, after receiving 6 Gy of irradiation, tumor volume ratios decreased in both groups. This suggests that reducing CD44 expression may increase the sensitivity of Cap cell lines to radiation (55). In a study published in 2010, Kong Z and colleagues presented evidence suggesting that DAB2IP-deficient prostate cancer cells that have metastasized show greater clonogenic survival when treated with ionizing radiation than control cells expressing normal levels by DAB2IP. This resistance is primarily attributed to the faster repair kinetics of DNA double-strand breaks in DAB2IP-deficient cells. All cells were subjected to a radiation dose of 3.47 Gy/min. The researchers eliminated endogenous DAB2IP from a metastatic prostate cancer cell line using the shRNA-lentiviral system and performed Western blot analysis to confirm the loss of DAB2IP protein expression (56). Kong Z and colleagues present evidence suggesting that DAB2IP-deficient prostate cancer cells that have metastasized show greater clonogenic survival when treated with ionizing radiation than control cells expressing normal levels of DAB2IP. This resistance is primarily attributed to the faster repair kinetics of DNA double-strand breaks in DAB2IP-deficient cells. All cells were subjected to a radiation dose of 3.47 Gy/min. The researchers eliminated endogenous DAB2IP from a metastatic prostate cancer cell line using the shRNA-lentiviral system and performed Western blot analysis to confirm the loss of DAB2IP protein 6 Following this study, in 2012, Yu L et al. found that the use of a novel DNA-PKcs inhibitor called NU7441 had a significant impact on the effect of radiation in DAB2IP-deficient PCa cells. The cells showed increased sensitivity to radiation after treatment with NU7441, mainly due to the delay in the repair of DNA double-strand breaks. This result highlights the potential of NU7441 as a valuable tool for increasing the efficacy of radiotherapy in prostate cancer patients (57). In 2016, another research conducted by Yang C et al. emphasized the importance of DAB2IP, supporting previous findings that loss of expression of disabled homologous interactive protein 2 (DAB2IP) in the normal prostatic epithelium and in PCa leads to resistance to γ-rays. To investigate the relationship between DAB2IP and ionizing radiation, PCa cells were subjected to 12 fractional irradiations of 2 Gy of γ-rays and DAB2IP mRNA expression was monitored. The study showed that DAB2IP expression levels continued to decline, indicating that radiotherapy-induced downregulation of DAB2IP may contribute to acquired radioresistance in PCa patients.

In 2013, Cintra HS and colleagues conducted a study aimed at evaluating the link between ATM, TP53 and MDM2 polymorphisms in prostate cancer patients undergoing external radiation therapy. The study involved forty-eight patients enrolled between January 2009 and December 2010. Treatment involved the use of high-energy photons (15 MV) and a daily dose of 2 Gy. The results revealed that clinical features do not play a significant role in disease sensitivity in prostate cancer patients. Instead, intronic polymorphisms of TP53 were associated with increased acute and chronic radiation toxicity (58).

The incorporation of genetic and genomic assessments into the management of prostate cancer marks a substantial advancement in the personalization of treatment regimens. These tests enhance the capacity to identify individuals at risk and furnish indispensable data for the development of more precise and efficacious treatments. Despite the necessity for further investigation to fully elucidate the potential and constraints of these instruments, their deployment is poised to markedly enhance clinical outcomes for prostate cancer patients.

All the studies are summarized in **Table 6**.

**Table 6:** Summary of studies investigating the use of genetic profiling in prostate cancer radiotherapy.

First Author	Year	Aim	Results
Citra HS	2013	to evaluate the association between ATM, TP 53 and MDM 2 polymorphisms in patients with prostate cancer and morbidity after radiotherapy.	Research suggests that intronic TP53 polymorphisms may be linked to higher rates of acute and chronic radiation toxicity
But JW	2018	whether co-expression of CD44 and ERBB2 was involved in CaP cell radioresistance and its mechanism	During observation, no changes in tumor volumes of control and CD44 knockdown cell lines before radiation exposure were detected.
Kong Z	2010	Understand the response to ionizing radiation (IR) of DAB2IP-deficient prostate cancer cells	DAB2IP has a crucial function in promoting prostate cell survival after IR exposure.
Yu L	2012	Show a novel function of DAB2IP in suppressing IR-induced and DNA-PKcs-associated autophagy and promoting apoptosis in PCa cells.	A novel DNA-PKcs inhibitor NU7441 can significantly enhance the effect of radiation in DAB2IP-deficient PCa cells
Yang C	2016	To observe whether decreased DAB2IP gene expression is associated with resistance to γ-rays and α-particles in prostate cancer cells	Radiotherapy-induced DAB2IP downregulation may be associated with acquired radioresistance in patients with PCa

## Cervical cancer

Every year, about 500,000 women worldwide are affected by cervical cancer, the 4° deadliest type of cancer.

Radiation therapy (RT) is a common treatment for cervical cancer, along with surgery. In the 2009 International Federation of Gynecology and Obstetrics (FIGO) staging, stage IB cervical cancer can be cured with chemoradiation therapy (CRT), with a 5 year local control rates of 98% for IB1 and 92% for IB2.

While women diagnosed with locally advanced stage have a five-year survival rate of less than 50%.

Cervical cancer screening by combined cytology and HPV testing has reduced the incidence of the cancer, but cytology screening has no higher sensitivity and HPV testing has a lower specificity.

There are molecular models and biomarkers that might have information about disease resistance or possible improved response to treatments; this information could potentially help the clinical practice by making treatment ever more customizable.

The details of the analyzed studies are summarized in **Table 7**.

**Table 7:** Summary of studies investigating the use of genetic profiling in Cervical cancer radiotherapy.

Author	Year	Endpoint	Results
S. Sood	2014	Evaluate the gene promoter methylation profile to identify genes which could predict the response to CRT	Methylation of the promoters of the ESR1 and MYOD1 genes correlated with a better response to treatment
Carlos Contreras Romero	2022	Identify the methylation status of specific gene promoters with predictive potential to the CRT response.	Methylation of BRD9 promoter and CTU1 demethylation associated at a higher overall survival.Demethylation of DOCK8 associated with therapy-resistant patients
Tae Oike	2021	Find candidate mutation profiles to radioresistance and to estimate the underlying mechanisms.	Mutations in KRAS and SMAD4 associated at photon resistant tumors
Kyung Hwan Kim	2021	Obtain mechanistic insights into treatment resistance in cervical cancer.	100 genes over-regulated in resistant patients. 85 genes overexpressed in DCB patients
Masanori Someya	2021	Analyzing the expression of CD8, FoxP3, HLA-1, PD-L1, to find good prognosis factors	Better prognosis with high presence of T cells in tumor microenvironments. Better prognosis in cells expressing HLA-1, Poor prognosis in FoxP3-positive cells.
Xinlin Jiao	2019	tissues using methylation-specific PCR and qRT-PCR.	SEPT9 mRNA and protein expression is upregulated in cervical cancer tissues. SEPT9 promotes proliferation, invasion and migration of cervical cancer cells. It interacts with the HMGB1-RB axis, increasing resistance to irradiation.
Cristina S. Fjeldbo	2020	Evaluate how paired data of MRI-based hypoxia biomarkers and genes in cervical cancer could help predict response to chemoradiation treatment.	The combination of these data provided a significantly better prediction of PFS than a biomarker alone. It showed a large difference in PFS between more and less hypoxic tumours.

CRT, Chemoradiotherapy; MRI, magnetic resonance imaging; PFS, Progression Free Survival.

Evaluating the tumor genomes of 100 patients undergoing CRT, S. Sood et al. identified that methylation of the ESR1 and MYOD1 gene promoters correlated with better response to treatments and conversely, their non-methylation was associated with worse prognosis (59).

Analyzing the methylation profile of 92 patients Carlos Contreras-Romero et al. identified additional gene promoters related to response to treatments but also to resistance to CRT.

They noted that methylation of the BRD9 promoter and unmethylation of the CTU1 gene were present in all patients with complete response.

In contrast, the presence of the unmethylated DOCK8 promoter was present in all therapy-resistant patients.

They also associated the presence of BRD9 methylation and CTU1 nonmethylation with tumors <5cm and associated with stage II (60).

Evaluating the genetic profile of patients with recurrence post CRT of cervical cancer 1B, Tae Oike et al. showed that the presence of simultaneous KRAS and SMAD4 mutations resulted in radioresistance not observed in other nonresistant tumors.

They next treated this sample *in vitro* with carbon ion radiotherapy noting a good response.

These results could point toward the use of this specific RT technique when simultaneous KRAS and SMAD4 mutations are present in cervical cancer (61).

Analyzing the gene difference between CRT-resistant patients with one other with durable clinical benefit (DCB)patients Kyung Hwan Kim et al. identified 185 differentially expressed genes (DEGs), of these, 100 genes were found to be significantly overexpressed in resistant patients and 85 genes were found to be significantly overexpressed in DCB patients. While the genes over-regulated in resistant patients were associated with extracellular matrix production, the genes over-regulated in DCB patients were associated with epidermal cell differentiation and interferon production. In addition, a high fraction of cancer-associated fibroblasts (CAF) was detected within the resistant tumors compared with DCB patients (62).

Xinlin Jiao et al. evaluated SEPT9 methylation and mRNA expression in various cervical tissues using methylation-specific PCR and qRT-PCR (63). They subsequently investigated the biological function and radiation resistance of SEPT9 *in vitro* and *in vivo*. The researchers found that SEPT9 mRNA and protein expression was upregulated in cervical cancer tissues compared to paracarcinoma tissues. SEPT9 promotes proliferation, invasion, and migration and affects the cell cycle of cervical cancer cells. It interacts with the HMGB1-RB axis, increasing resistance to irradiation. Additionally, SEPT9 mediates miR-375 through tumour-associated macrophage polarization (TAM), which influences resistance to radiotherapy in cervical cancer. These results demonstrate that SEPT9 methylation could be a biomarker for cervical cancer diagnosis. It promotes tumorigenesis and radioresistance of cervical cancer and could become a screening and therapeutic biomarker for cervical cancer.

Recent studies on biomarkers and genetic profiling offer promising avenues for the personalization of treatments, with the potential to enhance therapeutic response and prognosis. It is imperative that further research be conducted in this field in order to develop more effective screening strategies and treatments, with the ultimate goal of significantly improving clinical outcomes for patients diagnosed with cervical cancer.

## Discussion and future directions

The complex landscape of genetic profiling in radiotherapy presents a promising avenue for improving cancer treatment outcomes. The studies summarized across various cancer types highlight the potential of genomics in predicting radiosensitivity, guiding treatment decisions, and enhancing overall survival. However, it is important to acknowledge the current limitations and challenges in this field. One notable limitation is the lack of prospective trials that specifically investigate the role of genomic profiling in radiation therapy. While retrospective studies have provided valuable insights, prospective trials are needed to establish robust evidence for the clinical application of genetic biomarkers in radiotherapy. Data from retrospective studies could be the starting point for setting the organizational model and the objectives of prospective studies necessary to establish solid evidence of the clinical application of genetic biomarkers in radiotherapy. Such trials would help validate the predictive power of identified genetic variations and molecular signatures, ensuring that these biomarkers have meaningful clinical relevance. Another challenge lies in our incomplete understanding of the molecular mechanisms underlying radiotoxicity and radiosensitivity. The complexities of radiation response involve a multitude of genetic and molecular factors, making it difficult to identify a single universal biomarker for treatment response. To address this, further research is required to elucidate the intricate interactions between genetic variations, cellular pathways, and radiation response to develop more accurate predictive models. While deintensification of treatment is an essential goal in cancer management, it should be approached with caution. Genetic profiling may offer opportunities to identify patients who can safely receive reduced treatment intensity, potentially sparing them from unnecessary side effects. However, it is crucial to recognize that each patient’s case is unique, and a personalized approach that considers the entire spectrum of treatment options (chemotherapy, surgery, and radiotherapy) should be pursued. Precision medicine demands comprehensive evaluation of the patient’s clinical and genomic characteristics to ensure optimal treatment decisions.

In conclusion, the studies presented in this review represent the current state of the art of clinical research in radiotherapy and showcase the potential of genetic profiling in radiotherapy for different cancer types. These findings open up new possibilities for personalized treatment strategies that may improve patient outcomes and reduce treatment-related toxicity. Nonetheless, the field of radiogenomics still faces challenges in terms of prospective validation and a deeper understanding of the molecular mechanisms at play. Future research efforts should be directed towards conducting well-designed prospective trials and further unraveling the complexities of radiotoxicity and radiosensitivity to fully harness the potential of genetic profiling in guiding precision radiotherapy approaches.

In this scenario, radiomics also plays an important role in providing prognostic and predictive information to support clinical decision making (64).

Radiogenomics and Radiomics represent the current challenges in the assessment of radiation – induced toxicities, which could support a tailored radiation treatment management.

## Author contributions

DR: Data curation, Formal analysis, Methodology, Resources, Writing – original draft. FG: Data curation, Investigation, Methodology, Writing – original draft. VSM: Methodology, Supervision, Writing – original draft. LD: Data curation, Investigation, Methodology, Supervision, Writing – original draft. PG: Data curation, Investigation, Methodology, Writing – original draft. ID: Data curation, Investigation, Supervision, Writing – review & editing. ARP: Supervision, Writing – review & editing, Investigation. AS: Supervision, Writing – review & editing, Methodology. GR: Supervision, Validation, Writing – review & editing, Visualization. SC: Supervision, Writing – review & editing, Validation, Methodology, Project administration. VN: Conceptualization, Formal analysis, Funding acquisition, Supervision, Validation, Writing – original draft, Writing – review & editing. AR: Conceptualization, Funding acquisition, Investigation, Resources, Supervision, Writing – review & editing.
